# Flame Retardancy and Thermal Behavior of Wool Fabric Treated with a Phosphorus-Containing Polycarboxylic Acid

**DOI:** 10.3390/polym13234111

**Published:** 2021-11-25

**Authors:** Huaifang Wang, Shengnan Guo, Chuanjie Zhang, Zhichuang Qi, Lianfeng Li, Ping Zhu

**Affiliations:** 1 College of Textile and Clothing, Institute of Functional Textiles and Advanced Materials, College of Textile and Clothing, State Key Laboratory of Bio-Fibers and Eco-Textiles, Qingdao University, Qingdao 266071, China; hfwang1980@163.com (H.W.); gsn15726230106@163.com (S.G.); qzc2000qi@163.com (Z.Q.); 2 Shandong Ruyi Wool Textile Garment Group Co. Ltd., Jining 272073, China; ruyillf0828@163.com

**Keywords:** PBTCA, flame retardancy, thermal stability, wool, crosslinking, phosphorus, char forming

## Abstract

The compound 2-phosphonobutane-1,2,4-tricarboxylic acid (PBTCA) is an eco-friendly water treatment agent possessing flame-retardant phosphorus element and multi-carboxylic acid groups in its molecular structure. In the present work, PBTCA is employed as a finishing agent to improve the flame retardancy of the wool fabrics by the pad-dry-cure technique. The treated wool (10.2% weight gain) by 100 g/L of PBTCA showed an increased flame retardancy with a limiting oxygen index value (LOI) of 44% with a minimum char length of 40 mm. Importantly, the treated wool can self-extinguish after 30 washing cycles. The PBTCA-treated wool exhibited better stability with obviously increased char residue of 39.7% and 28.7% at 600 °C, while only 25.9% and 13.2% were measured for the control wool in nitrogen and air atmosphere, respectively. In addition, the high thermal stability of the treated wool with astonishing char-forming ability is confirmed by the SEM images of the wool after the isothermal heating treatment at different temperatures. Finally, a two-stage flame-retarding mechanism of enhanced crosslinking and char formability of PBTCA-treated wool is proposed and analyzed by infrared spectroscopy (TG-FTIR) and thermal (DSC and TGA) results of the pyrolytic volatiles of the treated wool.

## 1. Introduction

As one of the most essential natural protein fibers, wool is widely used in apparel, interior textiles, and industrial applications due to its superior aesthetic qualities along with excellent breathability, warmth, elasticity, and especially, the inherent low flammability. In woolen fabrics, the high amount of nitrogen, sulfur, and moisture contents are responsible for the intrinsic flame retardancy with a limiting oxygen index (LOI) of ≈25% [1,2]. However, the wool fabrics, which are not of very dense construction and heavy, still cannot pass the vertical burning test [3,4]. Therefore, it is necessary to enhance the flame retardancy of the wool.

In the past decades, various flame retardants have been developed to improve the flame retardancy of wool fabrics [2,5,6,7]. The halogen-based compounds including chloro- and bromo- derivatives had been investigated and coated to develop high efficiency and durable wool [2]. Nowadays, the halogen-containing flame retardants are rarely used due to releasing out of toxic dioxins during the burning process [2,8]. From the 1970s, the mordanting treatment of wool using zirconium/titanium complexes and their modified compounds has been investigated extensively [9,10]. Based on these findings, the famous “Zirpro” process was developed for making a flame-retardant wool with excellent washing durability. However, the effluents produced during the process have been criticized for the heavy metal pollution [11,12], and the treated wool turned yellow and lost its flame resistance if exposed to light or washed in alkaline solution [13]. The phosphorus-based compounds also received significant attention in the exploitation of various flame retardant polymers including wool fabrics for their low toxicity and high efficiency [5]. Notably, the two durable flame-retardant systems, namely Proban and Pyrovatex CP, which were designed and commercially used for cotton fabrics over the decades, were attempted on the wool fabrics. Although imparting a high degree of flame retardancy, the two systems were finally overlooked for releasing the toxic formaldehyde during the preparation of the flame-retardant fabrics and end-use process [2,14,15,16]. A vinyl phosphate was also applied on the wool by the graft method, demonstrating high flame retardancy with LOI above 35, but the tensile strength of the grafted wool was reduced sufficiently and needed to be improved [12]. Recently, the enhancement of flame resistance of the wool fibers has been focusing on the usage of sustainable flame retardants. The plant-based bio-molecules, for instance, banana pseudostem sap (BPS) and green coconut shell extract (CSE), have been investigated for enhancing flame retardancy in the protein wool owing to the content of phosphate, phosphite, nitrogen, silicates, and some metallic salts, endowing the self-extinguishable property and imparting coloration in the fabric as well [13,17,18].

Furthermore, a bio-based product of high phosphorus content, phytic acid (PA), has been investigated as well to confer flame resistance in the wool [6,19,20]. Additionally, a few organic compounds containing sulfur, boron, and silicon were tested as flame retardants for wool [21,22,23,24]. Some nano-based treatments have also been carried out to achieve flame-retardant wool [19,25]. Despite the excellent flame retardancy, most of these approaches are not reliable under the repeated washing due to the lack of covalent bonds between the flame retardant and wool keratin polypeptide. In this direction, various additives have been explored to improve water durability [19,21,26,27]. Among them, the introduction of polycarboxylic acids as the crosslinking agent, for example, 1,2,3,4-butanetetracarboxylic acid (BTCA), is verified as an effective organic compound to build the covalent connection between the flame retardant and wool [19,28].

**Figure 1:**
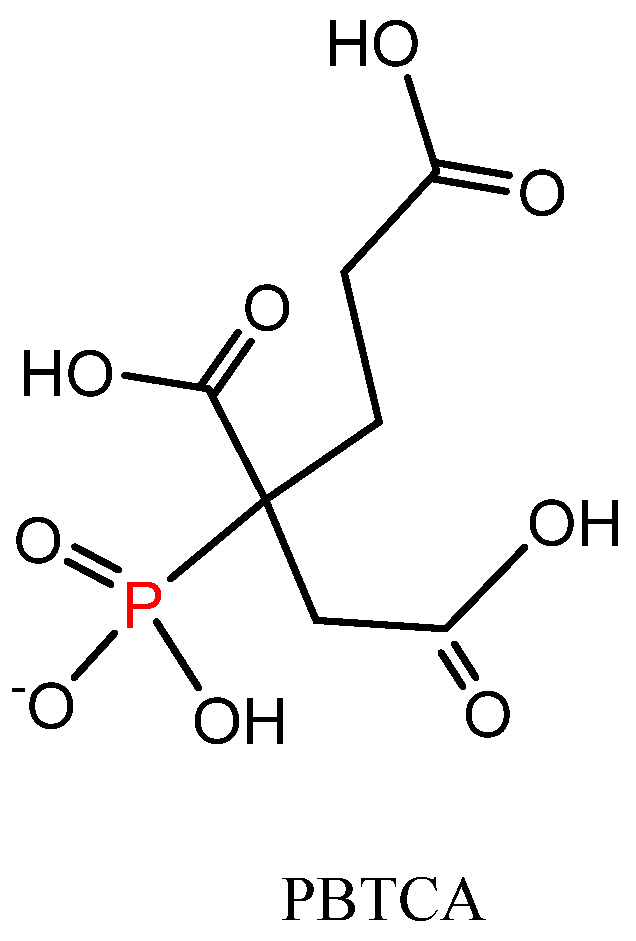
The chemical structure of PBTCA.

Based on the above-mentioned findings, an environmental-friendly and bio-degradable phosphorus-containing polycarboxylic acid (2-phosphonobutane-1,2,4-tricarboxylic acid, PBTCA) [29] aroused our interest. The chemical structure of PBTCA is shown in Figure 1. As presented, this compound is composed of the phosphorus element, which was regarded as having flame-retarding property, and multi-carboxyl groups, which has been found to be able to crosslink with hydroxyl (-OH) groups in cellulose [30,31] and -OH and/or amino (-NH_2_) groups in keratin chains in the wool [19,28]. Initially, PBTCA has been widely explored as an effective scale inhibitor, dispersant, and corrosion inhibitor due to its excellent chelating property [29,32,33]. Moreover, PBTCA has been examined as an anti-wrinkle agent for cotton fabrics as it possesses the structural character of polycarboxylic acids, forming ester crosslinking with -OH groups in the cellulose [34,35].

In the present work, we employed the PBTCA on the wool fabrics to investigate the flame retardancy and thermal behavior of the treated fabrics. The study is carried out by considering the weight loss analysis during heating of the wool treated by PBTCA in the counterparts of other phosphorus-containing chemicals. A slight weight loss is observed in the wool treated by PBTCA during the first decomposition process, while comparatively more weight loss is found in the other flame retardants. To get in depth into the flame-retardant mechanism and physical properties of the wool, the treated wool fabrics are examined using the thermo-analytical method, isothermal heat treatment, and infrared analysis.

## 2. Experimental

### 2.1. Materials

The woven wool fabric (147 g/m^2^, 102S/2 ∗ 54S) was supplied by Shandong Ruyi Wool Textile Garment Group Co. Ltd., Jining, China. The sodium hydroxide (NaOH) was purchased from Xilong Science Co. Ltd., Shantou, China. The anhydrous sodium carbonate (Na_2_CO_3_), sodium hypochlorite (NaClO), sulfuric acid (H_2_SO_4_), and anhydrous sodium sulfite (Na_2_SO_3_) were purchased from Sino-pharm Chemical Reagent Co. Ltd., Shanghai, China. All the reagents were used as the reagent grade without any further purification process. The compound, 2-phosphonobutane-1,2,4-tricarboxylic acid (PBTCA, 50% (*w*/*w*) aqueous solution), was purchased from Taihe Water Treatment Technologies CO. Ltd., Zaozhuang, China.

### 2.2. Fabric Treatment Procedure

#### 2.2.1. Wool Pretreatment

Basically, the wool fiber is covered by one or two layers of the cuticle, and the cuticle is surrounded by a fatty acid layer of 18-methyleicosanoic acid (18-MEA). The layer of 18-MEA is covalently bonded to the epicuticle through thioester bonds, which are together attached with the scaly surface, causing a water-repellent surface and a barrier to chemical treatment [36]. As demonstrated in the previous research, the partial removal of the scale layer by oxidation can assist the chemical diffusion into the fiber interior [37]. Therefore, in this work, the pretreatment of the wool was carried out to promote the diffusion of the finishing agent. Firstly, the raw wool fiber was treated in a solution containing NaClO (0.2% active chlorine, pH = 3) for 15 min at 25 °C. Thereafter, the chlorinated wool passed through the solution containing 0.1 wt % Na_2_CO_3_ and 0.05 wt % Na_2_SO_3_ at 40 °C and pH 9.0 for 40 min. So, the pretreated wool was used for further experimental steps as control wool.

#### 2.2.2. Flame-Retardant Treatment

The pad-dry-cure method was used for the flame-retardant treatment of wool fabrics. The pretreated wool fabric was first immersed in the aqueous PBTCA solution of various concentrations (20 g/L, 40 g/L, 60 g/L, 80 g/L, and 100 g/L at pH of 1.43~0.93) for 10 min at room temperature. Then, the padded process using a two-roll laboratory padding machine was carried out. The wet pick-up from the fabric was 100 ± 5% after two dips and two nips. Afterward, the padded fabric was dried in an oven at 80 °C for 3 min and subsequently cured at 170 °C for 3 min. At the end of the treatment, the wool fabric was rinsed with distilled water and dried at room temperature. The samples finished with the PBTCA of different concentrations were termed as W20, W40, W60, W80, and W100, respectively.

### 2.3. Characterization

#### 2.3.1. Weight Gain

The wool fabrics were dried in the oven at 100 °C for 30 min and then measured and weighed quickly. The weight gain of the treated wool was determined according to Equation (1):

(1) $$WG=\frac{W_{i}-W_{0}}{W_{0}}\times 100$$

where *W_i_* and *W*_0_ denote the weight of the wool fabric before and after treatment, respectively.

#### 2.3.2. LOI Test

The LOI values of the wool fabrics were recorded according to GB/T 5454-1997 (equivalent to ASTM D2863) on an oxygen index tester (LFY-606 B, Shandong Textile Science Research Institute, Qingdao, China). For each sample, the treated wool fabric was cut in the size of 150 mm × 58 mm for testing.

#### 2.3.3. Vertical Burning Testing (VBT)

The VBT was conducted according to GB/T 5455-2014 (equivalent to ASTM D6413) standard by using a vertical combustion apparatus (LFY-601 A, Shandong Textile Science Research Institute, China). The size of the sample was 300 mm × 80 mm, and the ignition time was 12 s. The values of the obtained char length for each sample were measured carefully.

#### 2.3.4. Washing Durability

To assess the resistance against the water of the treated wool fabrics, the treated samples were impregnated in the aqueous solution containing 0.15 wt % of AATCC 193 standard detergent at 45 °C for a specific period. The washing duration of 45 min was called 5-cycles washing. Thus, the fabric was taken out after 5, 10, 15, 20, and 25 washing cycles, and subsequently, the fabric was rinsed with distilled water and dried in the oven at 80 °C for 1 h. The flame retardancy of the washed wool samples was assessed with LOI.

#### 2.3.5. Attenuated Total Reflection Fourier Transform Infrared (ATR-FTIR) Spectroscopy

The chemical structure of the control wool and PBTCA-finished wool were analyzed by ATR-FTIR spectroscopy by a Nicolet iS 50 FTIR spectrometer (Thermo Fisher Scientific, Waltham, MA, USA) within the range of 500–4000 cm^−1^ with a resolution of 4 cm^−1^.

#### 2.3.6. Cone Calorimetric Test (CCT)

The combustion behavior of the wool samples was measured according to the ISO 5660 standard with a cone calorimeter device (FTT007, Fire Testing Technology, East Grinstead, UK). The sample was prepared in the size of 100 mm × 100 mm. Three pieces of a sample were wrapped in aluminum foil and burned at a heat flux of 35 kW/m^2^. The assessment values such as the time to ignition (TTI), the heat release rate (HRR), the total heat release (THR), and other related data were measured.

#### 2.3.7. Thermogravimetric Analysis (TGA)

The thermal degradation properties of wool fabrics were carried out using a TG analyzer (STA6000, PerkinElmer, Walsham, MA, USA). Samples of about 5 mg were investigated under nitrogen and air atmosphere from 25 to 700 °C at a heating rate of 10 °C/min.

#### 2.3.8. Isothermal Heating Treatment and SEM Observation

To observe the effect of PBTCA on the thermal stability of wool fabrics before and after the finishing treatment, the scanning electron microscope (SEM) images of the samples after the isothermal heating treatment as per the previous report [38] were captured. The fabric samples of the size of 50 mm × 50 mm were heated in the muffle roaster for 10 min at 200 °C, 300 °C, 400 °C, 500 °C, and 600 °C, respectively. Afterward, the fabrics were observed using SEM (VEGA 3, TESCAN, Czechoslovakia) at an accelerating voltage of 10 kV.

#### 2.3.9. Differential Scanning Calorimeter (DSC) Testing

The DSC testing was conducted using control wool and the PBTCA-treated wool via a differential scanning calorimeter (DSC 4000, PerkinElmer, Walsham, MA, USA). The amount of the sample, nearly 6 mg, was placed in the crucible cell under the nitrogen gas flow at a rate of 50 mL/min. The heating rate was kept at 15 °C/min, and the observation was carried out in the temperature range 50–300 °C.

#### 2.3.10. TG-FTIR

Thermogravimetric analysis coupled with FTIR (TG-FTIR) was conducted on a PerkinElmer STA 6000 TG analyzer equipped with a PerkinElmer FTIR spectrometer (PerkinElmer, Walsham, MA, USA) in the temperature range 40–700 °C at 10 °C/min under nitrogen atmosphere. FTIR was set to be within the range of 500–4000 cm^−1^ with a resolution of 4 cm^−1^.

## 3. Results and Discussion

### 3.1. Flame Retardancy and Durability

**Figure 2:**
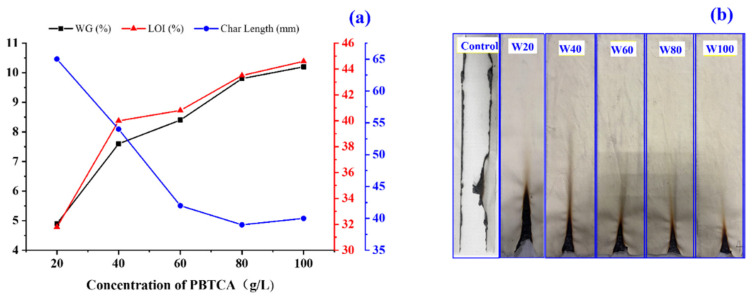
Flame retardancy of the wool fabrics treated with PBTCA (**a**) Weight gain, LOI, and char length of the control and treated wool samples and (**b**) Photos of the wool fabrics treated with different concentrations of PBTCA after VBT test.

In the present work, the vertical burning and LOI tests were carried out to determine the flame retardancy and the durability of PBTCA-treated wool samples. Figure 2 shows the weight gain, LOI, and VBT results of the wool treated with different concentrations of PBTCA. The control wool fabric showed an LOI of 24% and burned out completely during the vertical flammability tests, demonstrating a full char length of 300 mm. In comparison with the control wool, the wool fabrics treated with PBTCA show significantly higher LOI values and lower char length. With the increasing concentrations of PBTCA, the treated wool fabric acquires weight gain and shows improved flame-retardancy behavior, which is indicated by the increased LOI and decreased char length. The fabric finished with 100 g/L of PBTCA displayed an LOI of 44.6% and a decreased char length of 40 mm, respectively. Moreover, as compared to the control wool, the burning part of the PBTCA finished fabrics inflated obviously, showing the outstanding char-forming ability. The inflation of the charred wool is presented in the following CCT tests. These results indicated that the finishing of PBTCA can significantly improve flame retardancy in the treated wool.

**Figure 3:**
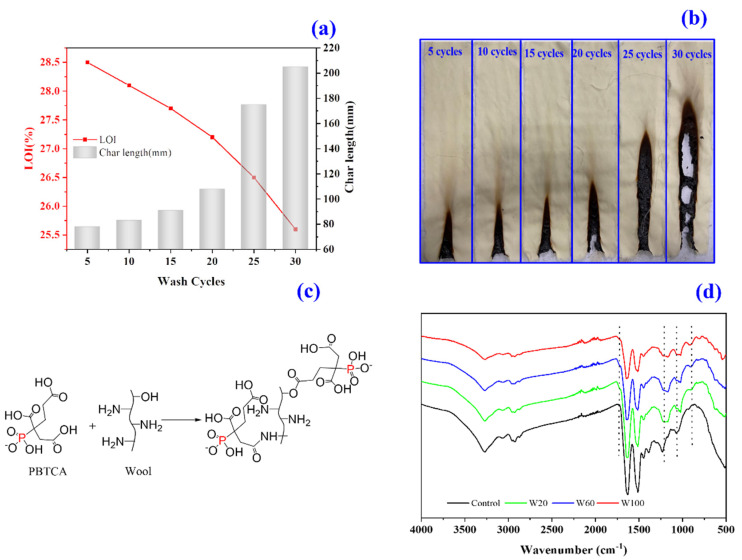
Washing durability of PBTCA-treated wool: (**a**) Char length of the wool treated with PBTCA (100 g/L) after repeated laundry cycles, (**b**) Photos of burned wool samples treated with PBTCA (100 g/L) after repeated washing cycles, (**c**) Possible crosslinking reaction between wool and PBTCA, (**d**) ATR-FTIR spectra of control wool and the PBTCA-treated wool, respectively.

The water durability of the PBTCA-treated fabrics was examined, and the results are illustrated in Figure 3a,b. With the increasing washing cycles, the treated wool fabrics show a reduced flame retardancy, which was verified by the increased char length and decreased LOI value. Specifically, even after 30 washing cycles, the treated wool fabrics still can sustain and self-extinguish in the burning process, showing a char length of 205 mm and LOI of 25.6. These findings indicated that the PBTCA-treated wool possessed good resistance to the laundry. According to the chemical structure of PBTCA and wool, some reactions may occur between the carboxyl groups of PBTCA and the -OH and/or -NH_2_ groups of the wool fabrics, as illustrated in Figure 3c. It is well verified by the obtained ATR-FTIR spectra of the treated wool, as shown in Figure 3d. In addition, the strong ionic bonds between the negative phosphate groups or carboxylate groups in PBTCA and the positive -NH_3_^+^ groups in wool may be attributed to the chemical reactions [19].

As shown in Figure 3d, the peaks that appeared at 1635 cm^−1^ and 1520 cm^−1^ in the samples of control wool, W20, W60, and Wl00 are the characteristic bands of amide I (C=O stretching) and amide II (N-H stretching) for wool fibers [27,39,40], respectively. In comparison with the spectrum of the control wool, the spectra of the treated samples displayed new peaks at 1230 cm^−1^ and 1060 cm^−1^, which are attributed to the vibration of P=O and PO^2−^ bonds from the phosphate groups in PBTCA [41]. A shoulder peak appeared at 1720 cm^−1^, which is ascribed to the stretching vibration of the ester carbonyl bonds and is possibly formed between the carboxylic acid in the PBTCA molecule and hydroxyl groups of serine, threonine, and tyrosine in wool fabrics, indicating the successful crosslinking reaction of PBTCA onto the wool fiber. As for the absorption of the amide through the carboxyl groups in PBTCA and amino groups in wool, it cannot be distinguished due to its overlapping by the original peptide adsorption from the wool.

### 3.2. Combustion Properties

**Figure 4:**
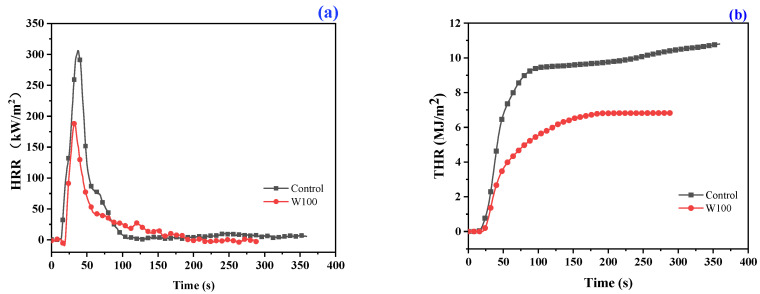

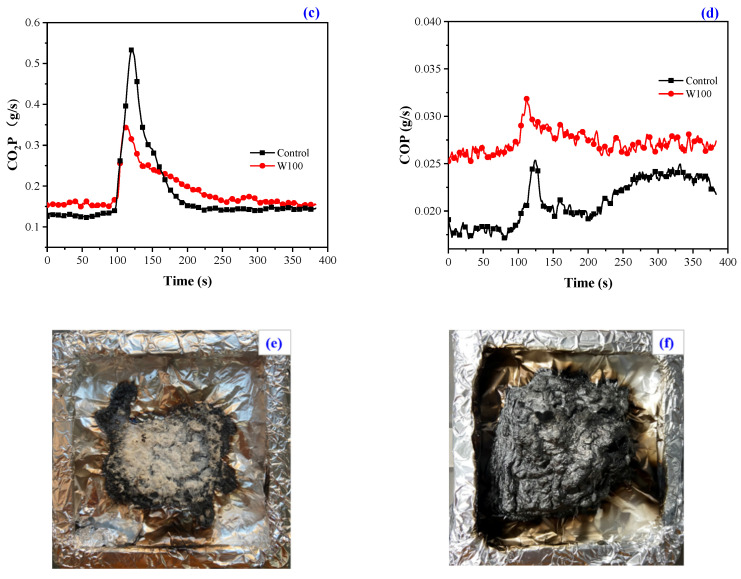
The HRR (**a**), THR (**b**), CO_2_P (**c**), and COP (**d**) curves of the control wool and the wool treated with PBTCA. Photos of the control (**e**) and the PBTCA-treated wool (**f**) after the cone calorimetry test.

The cone calorimetry test was employed to further investigate the flame retardancy of the treated wool under the heat flow. The THR and HRR of the control wool and PBTCA-treated (100 g/L) wool are shown in Figure 4. The obtained results such as the time to ignition (TTI), peak of heat release rate (PHRR), the average effective heat of combustion (Aver-EHC), the ratio of carbon dioxide (CO_2_) to carbon monoxide (CO), and the burnt residue after the test are presented in Table 1.

During the test, the PBTCA-treated wool underwent a less intensive burning behavior compared to the control wool after being ignited for a longer time. Burning of the treated wool fabric developed slowly and terminated in a comparatively longer time, releasing less heat. Specifically, as shown in Figure 4a,b and Table 1, the peak of the PHRR and THR of the PBTCA-treated fabric is 188.0 kW/m^2^ and 6.8 MJ/m^2^, respectively, which are much less than those values of the control wool (305.9 kW/m^2^ and 10.8 MJ/m^2^). In addition, the treated wool fabric turned into a significantly expanded intumescent char residue as compared to the control wool after the cone calorimetry test, as shown in Figure 4e,f. This type of char residue was beneficial to hinder the transfer of the heat and gaseous compounds between the outer and inner parts of the char covering the substrate, retarding the flame and suppressing the smoke production [18,39]. After the cone test, the remained char residue of 26.8% and 1.4% were measured for the treated wool fabric and the control wool, respectively, indicating that involving PBTCA on the wool can promote the char formability of the wool fabrics. The results can be further proved by the following TG analysis. Furthermore, as indicated in Figure 4c,d, the treated wool released a low CO_2_P and high COP amount. The treated wool fabric also showed lower Aver-EHC and higher CO_2_/CO than those values of the control wool, as illustrated in Table 1. The low Aver-EHC represented a flame inhibition effect induced by PBTCA on the wool during the combustion. The high CO_2_/CO suggested insufficient combustion and indicates that the introduction of this flame-retarding agent can hinder the combustion process in the treated wool [42], which is supposed to be attributed to the protection produced by the intumescent char layer during the combustion process.

The above-mentioned results of LOI, char length, and the cone calorimetry tests indicated that the treatment with PBTCA can enhance the flame retardancy of the wool fabrics by impeding the combustion and imparting the char formation reactions.

### 3.3. Analysis of Thermal Properties

**Figure 5:**
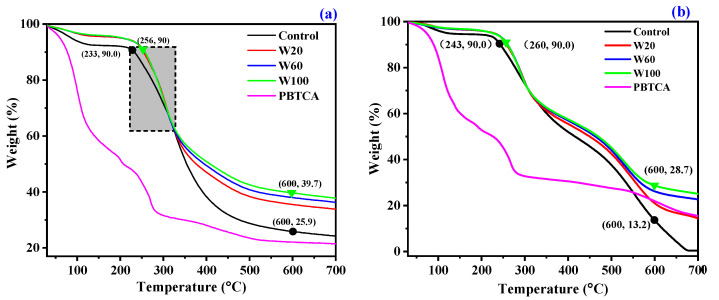
TG curves of the wool fabrics before and after treatment with PBTCA under nitrogen (**a**) and air (**b**) ambiance.

**Figure 6:**
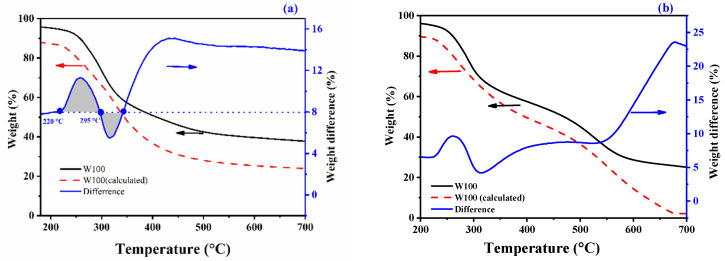
Difference between the actual and calculated TG curves of the PBTCA-treated wool in nitrogen (**a**) and air (**b**).

The thermal and thermal-oxidative stability of the PBTCA-treated wool fabrics were assessed by the TG analysis and compared to that of control wool. The obtained TG and DTG data of the control wool and the treated wool in nitrogen and air atmosphere are plotted in Figure 5 and Figure S1. The related thermal decomposition data are listed in Table 2. The comparison of the actual TG curves of the PBTCA-treated wool and the calculated curves (i.e., the curves calculated by the linear combination of the weight loss of wool and PBTCA) are displayed in Figure 6.

In the nitrogen, as can be seen from Figure 5a and Figure S1a, the wool fabrics underwent a degradation process of two main stages. The first stage takes place around 100 °C and corresponds to the desorption of the physically absorbed water. The second stage occurs above nearly 200 °C, where the major and sudden weight loss is observed. It is ascribed to breakage of the hydrogen bond in the peptide helical structure and to the change of the solid to liquid state of wool along with the cleavage of the disulfide bonds, causing the release of several volatile species, as demonstrated in the previous reports [6,43,44]. The PBTCA-treated wool exhibited higher thermal degradation temperature in the counterpart of the control wool, as indicated by the data listed in Table 2 and the values of T10% shown in Figure 5a. In addition, the introduction of PBTCA significantly enhanced the thermal stability of the treated wool, yielding more char residue. All the PBTCA-treated wool samples of W20, W60, and W100 exhibited higher residue char; for example, the value of the residue obtained from W100 is 39.7% at 600 °C in nitrogen, while the control wool just left a residue of 25.9%. It is quite clear that the residue difference between the treated wool and the control wool is larger than the value caused by the weight gain of the PBTCA itself, as indicated in Figure 6a. The actual TG curve of the PBTCA-treated wool indicated a much higher residue than the calculated one in the whole decomposition process. The calculated TG curve is based on a hypothesis of no chemical reaction occurring between the wool and the PBTCA. These results revealed that the degradation of PBTCA upon heating interferes with the degradation process of wool by favoring the char formation.

In the air, there are three stages during the pyrolysis process, as can be seen in Figure 5b and Figure S1b. The degradation curves follow the same general trend as that in nitrogen until 440 °C. The samples underwent a rapid decomposition above 440 °C in the air, which is related to the further oxidation of the remaining residue. The flame-retardant samples with high char residue exhibit the higher antioxidant ability of the formed char. Specifically, all the PBTCA-treated wool samples displayed a slower weight loss and higher residue in comparison with the untreated fabric, as shown in Table 2 and Figure 5b. For example, 28.7% of the residue was retained for the W100 sample at 600 °C, while only 13.2% was left for the untreated wool. Furthermore, with the increasing temperature, weight loss in the control wool increased dramatically; almost nothing was left at 700 °C. These findings were further verified by the “Difference” curve between the actual and calculated curves, as indicated in Figure 6b. The introduction of PBTCA significantly promotes the thermal stability of the formed char, which is even more obvious than its performance on the treated wool in nitrogen, as shown in Figure 6a. Thus, the significantly enhanced antioxidant ability of the formed char residue may be attributed to the enhanced crosslinking mechanism including the possible formation of P-N and P-O bonds for the introduction of phosphorus [43].

### 3.4. Isothermal Heat Treatment

**Figure 7:**
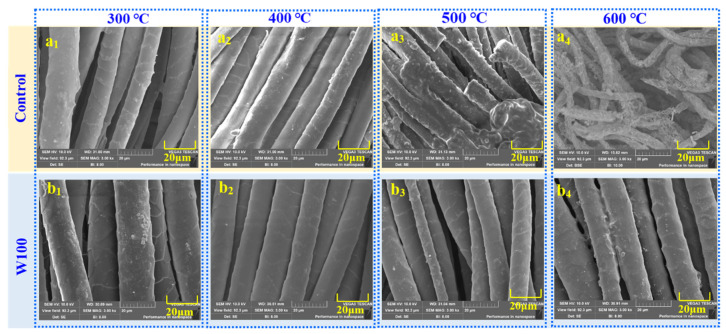
SEM of the control wool (**a_1_**–**a_4_**) and PBTCA-treated wool fabric (100 g/L, **b_1_**–**b_4_**) after being heated at different temperatures for 10 min.

To further observe the effect of the PBTCA on the thermal stability of the treated wool, the control and treated fabrics were heated in the muffle roaster at 200 °C, 300 °C, 400 °C, 500 °C, and 600 °C for 10 min, and subsequently, the surface morphology was observed by SEM, as shown in Figure 7. During the isothermal heating, both the control and PBTCA-treated wool carbonized after being heated at 300 °C for 10 min, and their surface appearance is much similar to that before and after treatment at 200 °C, as shown in Figure S2. As shown in Figure 7, the PBTCA-treated wool displayed better thermal stability, as there is no obvious change in the surface after being heated at the higher temperature (b_1_, b_2_, and b_3_) while the control fiber showed the swelling sign after being heated at 400 °C (a_2_) as the fiber became plumper than that at 300 °C (a_1_). Furthermore, a little amount of the control wool fibers broke and melted after being heated at 500 °C, and there was almost nothing left except for some porous ash for the control wool, as shown in Figure 7(a_4_). On the other hand, the PBTCA-treated wool after being heated at 600 °C for 10 min retained the good texture of the fabric and seemed to be covered by a thick coating, as shown in Figure 7(b_4_). It may be the poly-phosphorus acid layer formed from the pyrolysis of PBTCA and/or the melting and charring of the wool, which generate a compact char to retard the flame in the condensed phase and are helpful to restrain the smoke production.

Additionally, a smoke density test was performed to confirm this analysis, and the results are illustrated in Figure S3. In the whole burning process, much less smoke is generated from the PBTCA-treated wool than that from the control wool. The outstanding smoke-restraining property is ascribed to the formation of an intumescent char layer on the surface of the fabrics.

All the above-mentioned changes in the PBTCA-treated wool as compared with the control wool are well consistent with the results obtained from the TG test. The decomposition temperature shifted to a higher temperature after treatment with PBTCA, and the treated wool demonstrated a better char-forming ability. Therefore, the thermal stability of the PBTCA-treated fabric was greatly improved.

### 3.5. DSC

**Figure 8:**
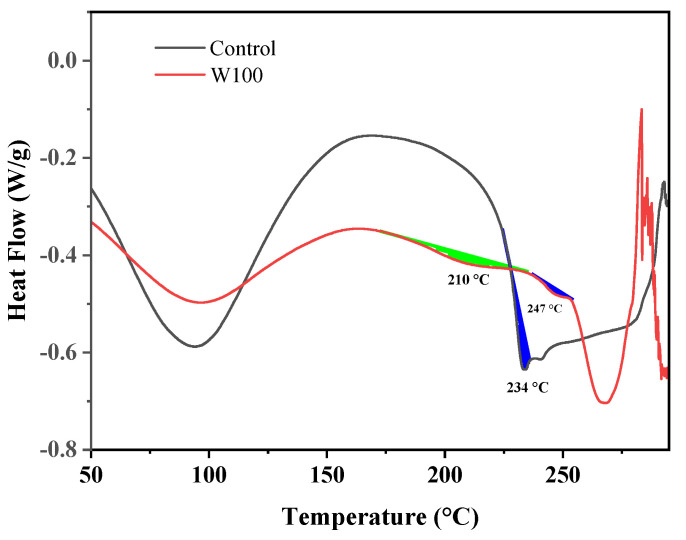
DSC curves of the control and PBTCA−treated wool.

Interestingly, the wool treated with PBTCA exhibited a slightly different decomposition trend below about 300 °C, as indicated by the curves in the dash frame in Figure 5a, in comparison with the effect of other phosphorus-containing compounds reported previously such as phosphoric acid [45], phytic acid [6,20], and ammonium phosphate compounds [39,43]. Specifically, the PBTCA-treated wool displayed an obviously lower and delayed weight loss. Similar results were also observed in the air atmosphere. We speculate that this phenomenon is caused by the enhanced crosslinking (upon heating) between peptide chains for the involved PBTCA, producing isopeptide or ester bonds, restraining the nature of the α-helix structure, changing phase from solid to liquid along with the rupture of disulfide bonds, and consequently, the thermal stability of the wool was improved [46]. In order to confirm this speculation, a DSC test was carried out for the control and PBTCA-treated wool, and the results are shown in Figure 8.

DSC is a powerful tool to investigate changes in the structure and the chemical damages in various keratins, modified keratins, and model keratin substances [47]. In the corresponding DSC curves, one or two endothermic peaks are observed in the temperature range 230–255 °C. Generally, wool is regarded as the composite of a microfibril–matrix structure, where a helical microfibril is embedded in an amorphous matrix of proteins associated with the microfibril. The first of the two peaks (peaks filled with blue color at 234 °C and 247 °C) for wool keratin is confirmed as a microfibrillar peak and assigned to an irreversible helix unfolding superimposed of the many decomposition reactions [47,48].

As per the previously reported literature, the temperature required for this denaturation process was found to be strongly affected by the crosslinking density in the matrix phase [49,50]. From Figure 8, the helix peak temperature increased from 234 to 247 °C, which supports the fact that the considerable stabilizing effects were produced possibly by enhanced crosslinking due to PBTCA rather than the disulfide crosslinking exclusively. A new endothermic peak (filled with green color) in the curve of the PBTCA-treated wool was observed at about 210 °C, which may be attributed to the reaction heat needed for the crosslinking between PBTCA and PBTCA and NH_2_ and/or OH groups of wool molecules. It is consistent with the esterification and acylation temperatures, as illustrated in Figure 3c. These observations support the explanation of the thermal stability of the PBTCA-treated wool, which can be ascribed to the enhanced crosslinking.

### 3.6. TG-FTIR

**Figure 9:**
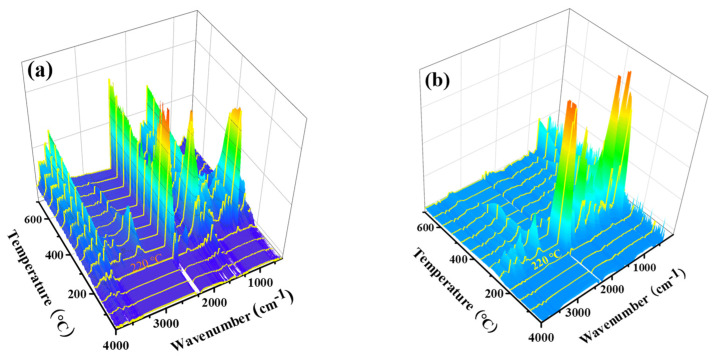

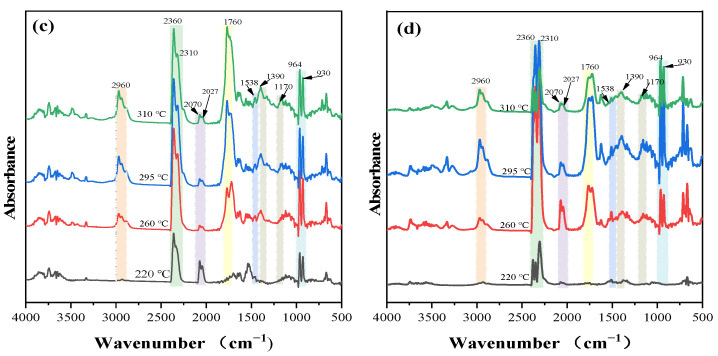
Three-dimensional (3D) TG-FTIR image and IR spectra of the control (**a**,**c**) and PBTCA-treated wool (**b**,**d**).

To investigate the flame-retardant mechanism, TG-FTIR was used to monitor the volatile pyrolysis products during the thermal degradation process in the nitrogen atmosphere. As shown in Figure 9, the 3D TG-FTIR images show the overall IR absorption of the pyrolytic volatiles produced from the control and PBTCA-treated fabric. FTIR spectra at four specific temperatures, namely 220 °C, 260 °C, 295 °C, and 310 °C, are presented in Figure 9c,d. The four temperatures were peaked from the range in the area of gray color in Figure 5a to verify and highlight the different decomposition processes due to PBTCA.

By comparing the 3D image and spectra of the control wool to that of the treated, it can be seen that similar volatile species were released from the two wool samples during the decomposition process. The bands at the range of 2830–3240 cm^−1^ correspond to the hydrocarbon volatiles [51]. The peaks at 2360 cm^−1^ and 2310 cm^−1^ are attributed to CO_2_, the peaks that appeared at 2070 cm^−1^ and 2027 cm^−1^ are ascribed to the absorption of CO, the peak at 1538 cm^−1^ is ascribed to the volatility of H_2_S, the peaks at 1390 cm^−1^ and 1170 cm^−1^ are attributed to the SO_2_, and the peaks at 964 cm^−1^ and 930 cm^−1^ are attributed to the NH_3_. It can be concluded that significantly fewer amounts of volatiles were produced from the PBTCA-treated wool (W100), as indicated in Figure 9b,d, especially in higher temperatures. A much more later decomposition took place for the PBTCA-treated sample, as almost no volatile was monitored until at 220 °C except for small CO_2_ absorption bands at around 2300 cm^−1^ (Figure 9b). In contrast, there were significantly more amounts of volatiles observed at wavenumbers around 3700 cm^−1^, 2300 cm^−1^, 1500 cm^−1^, and 1000 cm^−1^ in Figure 9a, which are attributed to the release of H_2_O, CO_2_, H_2_S, SO_2,_ and NH_3_. These findings also support the results from the DSC tests. The enhanced crosslinking of the isopeptide or ester bonds between peptide chains is caused by the introduction of PBTCA, reducing the decarboxylation and the amino groups breaking off from the peptide chains. Furthermore, the peaks assigned to sulfur-containing chemicals in the range of 1300–1550 cm^−1^ decreased dramatically for the PBTCA-treated wool, as displayed in Figure 9b.

### 3.7. Analysis of the Flame-Retardancy Mechanism

**Figure 10:**
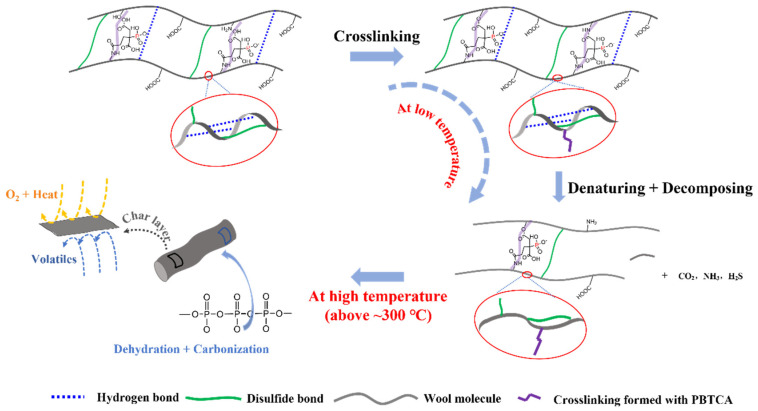
Flame-retardant mechanism of the PBTCA-treated wool at low and high temperature.

All the demonstrated results indicate that the incorporation of PBTCA can hinder the pyrolysis of the wool protein. Based on the above-mentioned analysis, the underlying mechanisms are proposed, as shown in Figure 10. In the low-temperature range, the introduction of the PBTCA can facilitate more isopeptide and/or ester bonds upon heating between the peptide chains, thereby improving the thermal stability of the treated wool. In addition, the enhanced crosslinking also restrains the release out of ammonia and CO_2_ caused by the side amide and carboxyl groups breaking off, showing less volatiles and delay decomposition for the PBTCA-treated wool. At high temperature (possibly above 300 °C as indicated in Figure 5 and Figure 6), the decomposition product of PBTCA can form a layer of polyphosphoric acid or diffuses into the wool, and the catalytic forming of a stable char layer occurred, suppressing the decomposition process of the treated wool and retarding it in the condensed phase, which is also confirmed by the EDS test results of PBTCA-treated wool after and before VBT, as shown in Figure S4. By comparing with the wool fabric before burning, the content of C and P showed an obvious increment, indicating an excellent carbonization ability of P in the condensed phase.

## 4. Conclusions

In summary, a phosphorus-containing polycarboxylic acid, PBTCA, was applied to the wool fabric. The flame retardancy (flammability and combustion behavior) of the treated fabrics turned out to be greatly enhanced by the incorporation of PBTCA. The flame retardancy behavior was sustained after repeated washing, which is because of the amide and ester connection between the PBTCA and wool. The TG results show that the thermal stability, oxidative resistance, and char-forming ability of the PBTCA-treated wool fabrics can be greatly improved, retaining 39.7% and 28.7% of the residue at 600 °C in the nitrogen and air atmosphere, respectively. The enhanced stability of the PBTCA-treated wool with better char-forming property and thermal degradation was also verified by the SEM images of the treated wool after being heated by isothermal treatment. The DSC and TG-FTIR tests were employed to explain plausible mechanisms related to the enhanced stability and flame retardancy of the treated wool. From the in-depth thermal analysis, a “two-stage” flame-retardant mechanism was proposed by the PBTCA, which can promote the crosslinking between the peptide chains in the low-temperature range and the char formation ability in the higher temperature range.

## Figures and Tables

**Table 1:** Results of cone calorimetry for the control wool and the PBTCA (100 g/L)-treated wool.

Samples	TTI (s)	PHRR (kW/m^2^)	Aver-EHC	CO_2_/CO	Residues (%)
Control	9	305.9	19.5	13.1	1.4
W100	14	188.0	14.8	17.4	26.8

**Table 2:** TG data of PBTCA-treated and control wool fabrics in nitrogen and air.

Samples		T_10%_^a^ (°C)	T_50%_^b^ (°C)	Residue at 700 °C (%)
N_2_	Control	233	354	24.2
	W20	253	376	33.8
	W60	256	394	36.3
	W100	256	408	37.7
Air	Control	241	415	0.36
	W20	256	450	14.3
	W60	259	463	22.6
	W100	259	469	25.1

^a^ Temperature at 10% weight loss; ^b^ Temperature at 50% weight loss.

## Data Availability

The data presented in this study are available in this article.

## Supplementary Materials

The following are available online at https://www.mdpi.com/article/10.3390/polym13234111/s1, Figure S1: DTG curves of the wool fabrics before and after treatment with PBTCA in nitrogen (a) and air (b); Figure S2. Picture (a) and SEM of wool before (a1,b1) and after (a2,b2) the isothermal heating treatment at 200 °C; Figure S3. The smoke density curves of the treated wool by the smoke density test; Figure S4. EDS results of the PBTCA-treated fabric before (a) and after (b) the VBT test.

## References

[1] Wortmann F.J., Eichhorn J.W.S.H.S.J., Jaffe M., Kikutani T. (2009). The structure and properties of wool and hair fibres. Handbook of Textile Fibre Structure.

[2] Horrocks A.R. (1986). Flame-retardant finishing of textiles. Rev. Prog. Coloration.

[3] Benisek L. (1984). Zirpro Wool Textiles. Fiber Mater..

[4] Kozłowski R.M., Muzyczek M., Kozłowski R.M., Mackiewicz-Talarczyk M. (2020). 10—Improving the flame retardancy of natural fibres. Handbook of Natural Fibres.

[5] Schartel B. (2010). Phosphorus-based Flame Retardancy Mechanisms-Old Hat or a Starting Point for Future Development?. Materials.

[6] Cheng X., Guan J., Chen G., Yang X., Tang R. (2016). Adsorption and flame retardant properties of bio-based phytic acid on wool fabric. Polymers.

[7] Basak S., Ali S.W. (2016). Sustainable fire retardancy of textiles using bio-macromolecules. Polym. Degrad. Stab..

[8] Carosio F., Di Blasio A., Alongi J., Malucelli G. (2013). Green DNA-based flame retardant coatings assembled through Layer by Layer. Polymer.

[9] Benisek L. (1974). Communication: Improvement of the natural flame-resistance of wool. Part I: Metal-complex applications. J. Text. Inst..

[10] Forouharshad M., Montazer M., Bameni Moghadam M., Saligheh O. (2010). Flame retardancy of wool fabric with Zirconium oxychloride optimized by central composite design. J. Fire Sci..

[11] Zhang X., Zhou X., Cheng X., Tang R. (2018). Phytic acid as an eco-friendly flame retardant for silk/wool blend: A comparative study with fluorotitanate and fluorozirconate. J. Clean. Prod..

[12] Zhang F., Guan J., Chen G. (2014). Performance of flame retardant wool fabric grafted with vinyl phosphate. J. Eng. Fiber Fabr..

[13] Basak S., Samanta K.K., Chattopadhyay S.K., Pandit P., Maiti S. (2016). Green fire-retardant finishing and combined dyeing of proteinous wool fabric. Color. Technol..

[14] Kaynak E., Üreyen M.E., Koparal A.S. (2019). Halogen free flame retardant finishing of wool and wool rich fabrics for aircraft seats. Mater. Today Proc..

[15] Michael E., Hall S.S. (1991). The Reaction of wool with N-Hydroxymethyl phosphonopropionamide. Polym. Degrad. Stab..

[16] Avraham B., Sarah H., Menachem L. (1979). The Chemistry of THPC-Urea polymers and relationship to flame retardance on wool and wool-polyester blends. II. Relative FlameRetardant Efficiency on Wool, Polyester, and Wool-Polyester Blends. J. Polym. Sci. Pol. Chem..

[17] Teli M.D., Pandit P. (2017). Novel method of ecofriendly single bath dyeing and functional finishing of wool protein with coconut shell extract biomolecules. ACS Sustain. Chem. Eng..

[18] Basak S., Raja A.S.M., Saxena S., Patil P.G. (2021). Tannin based polyphenolic bio-macromolecules: Creating a new era towards sustainable flame retardancy of polymers. Polym. Degrad. Stab..

[19] Cheng X., Guan J., Yang X., Tang R. (2018). Durable flame retardant wool fabric treated by phytic acid and TiO_2_ using an exhaustion-assisted pad-dry-cure process. Themochim. Acta.

[20] Cheng X., Guan J., Kiekens P., Yang X., Tang R. (2019). Preparation and evaluation of an eco-friendly, reactive, and phytic acid-based flame retardant for wool. Ract. Funct. Polym..

[21] Mathur P., Sheikh J.N., Sen K. (2020). Durable flame-retardant wool using sulphamic acid. Polym. Degrad. Stab..

[22] Cheng X., Zhang W., Wu Y., Ma Y., Xu J., Guan J. (2021). Borate functionalized caramel as effective intumescent flame retardant for wool fabric. Polym. Degrad. Stab..

[23] Shan G., Jia L., Zhao T., Jin C., Liu R., Xiao Y. (2017). A Novel DDPSi-FR Flame retardant treatment and its effects on the properties of wool fabrics. Fiber Polym..

[24] Zhang Q., Zhang W., Huang J., Lai Y., Xing T., Chen G., Jin W., Liu H., Sun B. (2015). Flame retardance and thermal stability of wool fabric treated by boron containing silica sols. Mater. Des..

[25] Jose S., Shanmugam N., Das S., Kumar A., Pandit P. (2019). Coating of lightweight wool fabric with nano clay for fire retardancy. J. Text. Inst..

[26] Cheng X., Tang R., Yao F., Yang X. (2019). Flame retardant coating of wool fabric with phytic acid/polyethyleneimine polyelectrolyte complex. Prog. Org. Coat..

[27] Cheng X., Guan J., Yang X., Tang R., Yao F. (2019). A bio-resourced phytic acid/chitosan polyelectrolyte complex for the flame retardant treatment of wool fabric. J. Clean. Prod..

[28] Martel B., Weltrowski M., Ruffin D., Morcellet M. (2002). Polycarboxylic acids as crosslinking agents for grafting cyclodextrins onto cotton and wool fabrics: Study of the process parameters. J. Appl. Polym. Sci..

[29] Yang B., Zhu Z., Yin W., Sun Q., Sun H., Han H., Sheng Q., Yao J. (2020). Selective adsorption of an eco-friendly and efficient depressant PBTCA onto dolomite for effective flotation of fluorapatite from dolomite. Chem. Eng. J..

[30] Yang C.Q., Wang X. (1986). Formation of Cyclic Anhydride intermediates and esterification of cotton cellulose by multifunctional carboxylic acids: An infrared spectroscopy study. Text. Res. J..

[31] Welch C.M. (1988). Tetracarboxylic acids as formaldehyde-free durable press finishing agents Part I: Catalyst, additive, and durability studies. Text. Res. J..

[32] Zhang B., Zhang L., Li F., Hu W., Hannam P.M. (2010). Testing the formation of Ca–phosphonate precipitates and evaluating the anionic polymers as Ca-phosphonate precipitates and CaCO_3_ scale inhibitor in simulated cooling water. Corros. Sci..

[33] Liu L., Cao T., Zhang Q., Cui C. (2018). Organic phosphorus compounds as inhibitors of corrosion of carbon steel in circulating cooling water: Weight loss method and thermodynamic and quantum chemical studies. Adv. Meter. Sci. Eng..

[34] Song H., Sui S., Zhu P., Dong Z., Zhang L. (2011). Wrinkle resistant finishing of cotton fabrics with the complex system of PBTCA/BTCA. Text. Aux..

[35] Wang H., Sheng G., Wang W. (2008). The discussion of wrinkle resistant finish of cotton and modified cotton fabric with PBTCA. Text. Aux..

[36] Hassan M.M., Leighs S.J. (2017). Effect of surface treatments on physicomechanical, stain-resist, and UV protection properties of wool fabrics. Appl. Surf. Sci..

[37] Hsieh S.H., Huang Z.K., Huang Z.Z., Tseng Z.S. (2004). Antimicrobial and physical properties of woolen fabrics cured with citric acid and chitosan. J. Appl. Polym. Sci..

[38] Davies P.J., Horrocks A.R., Miraftab M. (2000). Scanning electron microscopic studies of wool//intumescent char formation. Polym. Int..

[39] Liu Y., Guo Y., Ren Y., Wang Y., Guo X., Liu X. (2020). Phosphorylation of sodium copper chlorophyll enables color-fasten and durable flame retardant wool fibers. Polym. Degrad. Stab..

[40] Yin D., Wang J., Zhang X. (2001). Study of synthesis and structure of PBTCA. J. Dalian Univ. Technol..

[41] Alongi J., Carletto R.A., Di Blasio A., Carosio F., Bosco F., Malucelli G. (2013). DNA: A novel, green, natural flame retardant and suppressant for cotton. J. Mater. Chem. A.

[42] Zhao X., Babu H.V., Llorca J., Wang D. (2016). Impact of halogen-free flame retardant with varied phosphorus’s chemical surrounding on the properties of diglycidyl ether of bisphenol-A type epoxy resin: Synthesis, fire behaviour, flame-retardant mechanism and mechanical properties. RSC Adv..

[43] Horrocks A.R., Davies P.J. (2000). Char formation in flame-retarded wool fibres. Part 1. Effect of intumescent on thermogravimetric behaviour. FIRE Mater..

[44] Beck P.J., Gordon P.G., Ingham P.E. (1976). Thermogravimetric analysis of flame-retardant-treated wools. Text. Res. J..

[45] Ingham P.E. (1971). The Pyrolysis of wool and the Action of the flame retardant. J. App. Polym. Sci..

[46] Senoz E., Wool R.P., McChalicher C.W.J., Hong C.K. (2012). Physical and chemical changes in feather keratin during pyrolysis. Poly. Degrad. Stab..

[47] Spei R.H.M. (1990). Further thermoanalytical investigations of annealed keratins: The time and temperature dependence. Colloid Polym. Sci..

[48] Spei M., Holzem R. (1987). Thermo analytical investigations of extended and annealed keratins. Colloid Polym. Sci..

[49] Wortmann F.J., Springob C., Sendelbach G. (2002). Investigations of cosmetically treated human hair by differential scanning calorimetry in water. J. Cosmet. Sci..

[50] Wortmann F.J., Sendelbach G., Popescu C. (2007). Fundamental DSC investigations of α-keratinous materials as basis for the interpretation of specific effects of chemical, cosmetic treatments on human hair. J. Cosmet. Sci..

[51] Brebu M., Spiridon I. (2011). Thermal degradation of keratin waste. J. Anal. Polym. Pyrol..

