# Supplementary figures and images for: Flame Retardancy and Thermal Behavior of Wool Fabric Treated with a Phosphorus-Containing Polycarboxylic Acid
Source: Polymers (Basel). 2021 Nov 25;13(23):4111. doi: 10.3390/polym13234111 (PMC8659226; doi:10.3390/polym13234111)

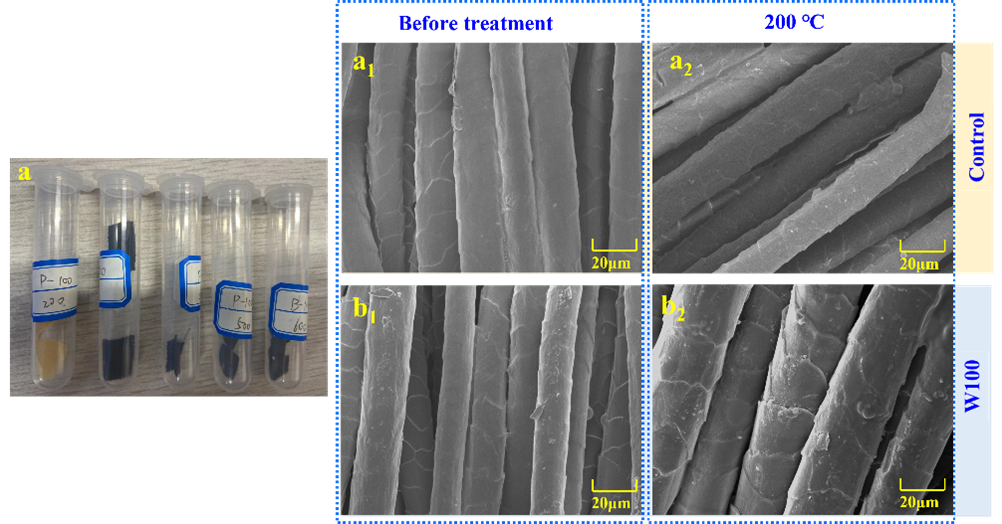

Supplement: Supplementary file 1 [file polymers-13-04111-s001.zip › Figure S2.tif]

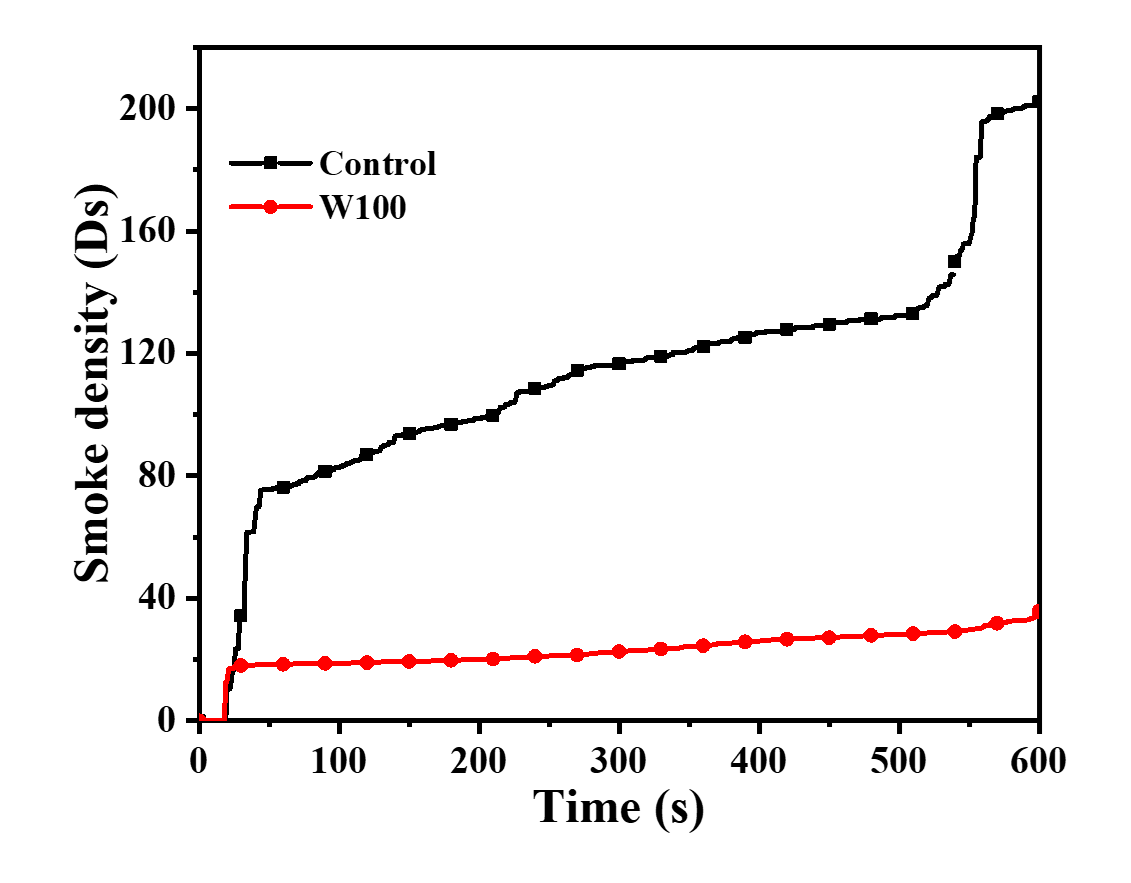

Supplement: Supplementary file 1 [file polymers-13-04111-s001.zip › Figure S3.tif]

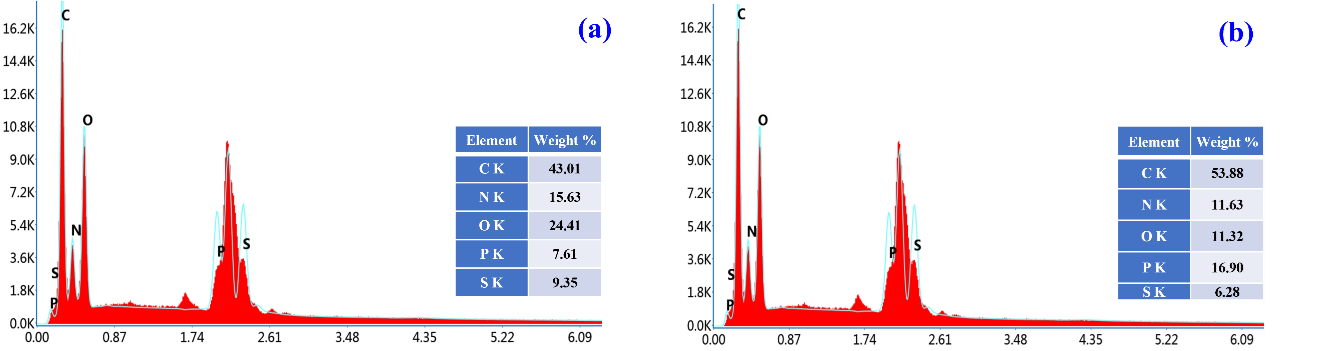

Supplement: Supplementary file 1 [file polymers-13-04111-s001.zip › Figure S4.tif]

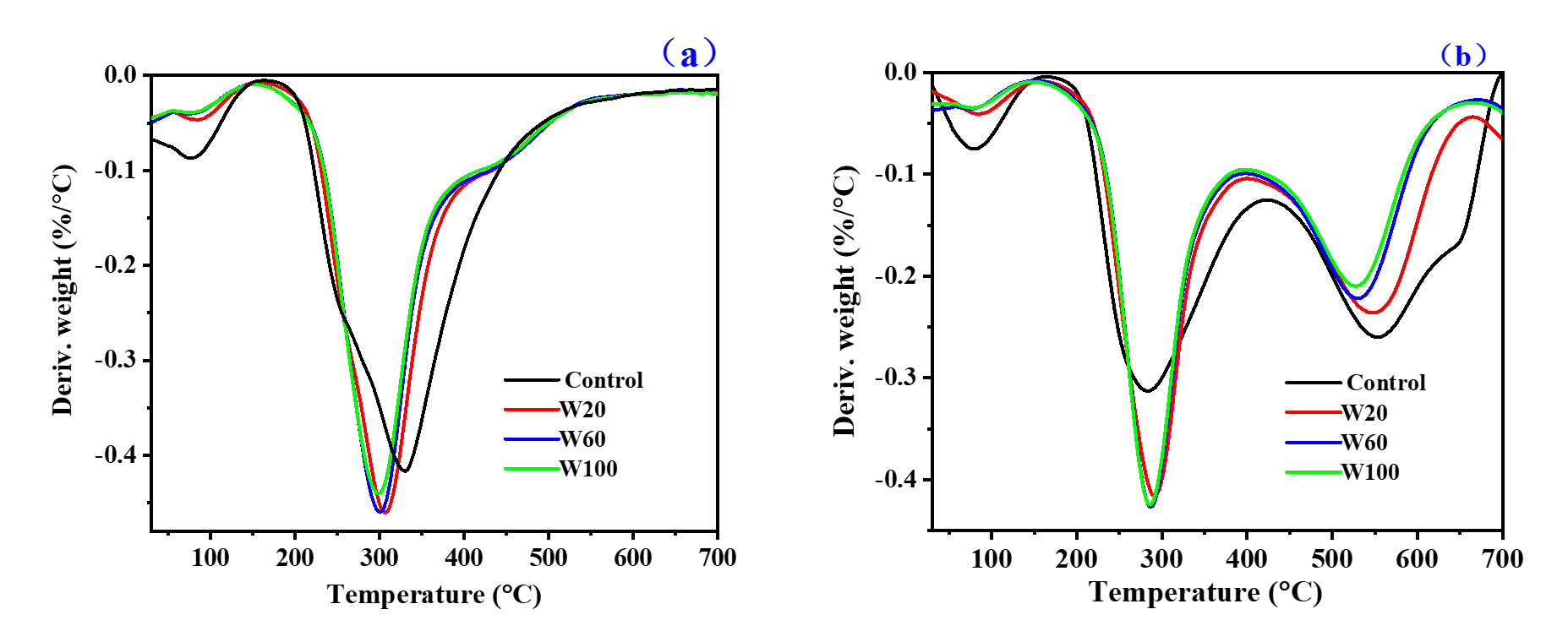

Supplement: Supplementary file 1 [file polymers-13-04111-s001.zip › Figure S1.tif]
